# Centralizing prescreening data collection to inform data-driven approaches to clinical trial recruitment

**DOI:** 10.1186/s13195-023-01235-4

**Published:** 2023-05-02

**Authors:** Dylan R. Kirn, Joshua D. Grill, Paul Aisen, Karin Ernstrom, Seth Gale, Judith Heidebrink, Gregory Jicha, Gustavo Jimenez-Maggiora, Leigh Johnson, Elaine Peskind, Kelly McCann, Elizabeth Shaffer, David Sultzer, Shunran Wang, Reisa Sperling, Rema Raman

**Affiliations:** 1 Department of Neurology, Brigham and Women’s Hospital, Harvard Medical School, Boston, MA USA; 2 Department of Neurology, Massachusetts General Hospital, Harvard Medical School, Charlestown, MA USA; 3 Institute for Memory Impairments and Neurological Disorders, University of California Irvine, Irvine, CA USA; 4 Department of Psychiatry and Human Behavior, University of California Irvine, Irvine, CA USA; 5 Department of Neurobiology and Behavior, University of California Irvine, Irvine, CA USA; 6 Alzheimer’s Therapeutic Research Institute, University of Southern California, San Diego, CA USA; 7 Department of Neurology, University of Michigan, Ann Arbor, MI USA; 8 Sanders-Brown Center On Aging, University of Kentucky, Lexington, KY USA; 9 Institute for Translational Research, University of North Texas Health Science Center, Fort Worth, TX USA; 10 VA Northwest Mental Illness Research, Education, and Clinical Center (MIRECC), VA Puget Sound Health Care System, Seattle, WA USA; 11 Department of Psychiatry and Behavioral Sciences, University of Washington School of Medicine, Seattle, WA USA; 12 Department of Neurology, Georgetown University Medical Center, Washington, D.C USA

**Keywords:** Alzheimer’s disease, Recruitment, Diversity

## Abstract

**Background:**

Recruiting to multi-site trials is challenging, particularly when striving to ensure the randomized sample is demographically representative of the larger disease-suffering population. While previous studies have reported disparities by race and ethnicity in enrollment and randomization, they have not typically investigated whether disparities exist in the recruitment process prior to consent. To identify participants most likely to be eligible for a trial, study sites frequently include a prescreening process, generally conducted by telephone, to conserve resources. Collection and analysis of such prescreening data across sites could provide valuable information to improve understanding of recruitment intervention effectiveness, including whether traditionally underrepresented participants are lost prior to screening.

**Methods:**

We developed an infrastructure within the National Institute on Aging (NIA) Alzheimer’s Clinical Trials Consortium (ACTC) to centrally collect a subset of prescreening variables. Prior to study-wide implementation in the AHEAD 3–45 study (NCT NCT04468659), an ongoing ACTC trial recruiting older cognitively unimpaired participants, we completed a vanguard phase with seven study sites. Variables collected included age, self-reported sex, self-reported race, self-reported ethnicity, self-reported education, self-reported occupation, zip code, recruitment source, prescreening eligibility status, reason for prescreen ineligibility, and the AHEAD 3–45 participant ID for those who continued to an in-person screening visit after study enrollment.

**Results:**

Each of the sites was able to submit prescreening data. Vanguard sites provided prescreening data on a total of 1029 participants. The total number of prescreened participants varied widely among sites (range 3–611), with the differences driven mainly by the time to receive site approval for the main study. Key learnings instructed design/informatic/procedural changes prior to study-wide launch.

**Conclusion:**

Centralized capture of prescreening data in multi-site clinical trials is feasible. Identifying and quantifying the impact of central and site recruitment activities, prior to participants signing consent, has the potential to identify and address selection bias, instruct resource use, contribute to effective trial design, and accelerate trial enrollment timelines.

## Background

The recruitment phase of multi-site clinical trials represents a large, but modifiable, component of total trial duration and cost [1, 2]. “Successful” recruitment for a trial includes not only accruing the sample on schedule [3] but also enrolling a sample that is representative of the larger disease-suffering population [4–7]. There are several known barriers to participating in clinical trials, particularly in historically underrepresented communities [8, 9]. Few interventions have been demonstrated to overcome these barriers [10].

The evaluation of recruitment strategies is generally focused on actual enrollment, including successful screening and randomization of study participants. However, measuring activity prior to trial enrollment may provide valuable information for both central and local efforts to accelerate and diversify recruitment, instruct resource expenditures, adjust recruitment campaigns, and, if needed, amend protocols to address observed selection bias.

Efforts to capture prescreening recruitment data may be particularly valuable in preclinical Alzheimer’s disease (AD) trials. These trials enroll cognitively unimpaired older volunteers who are screened for biological markers of AD [11]. Traditional clinical trial recruitment methods may not be effective in these trials, especially when the goal is to recruit a demographically representative cohort. In the first multi-site preclinical AD trial, the Anti-Amyloid treatment in Asymptomatic AD (A4) study [12], it was shown that participants from underrepresented racial and ethnic groups were more frequently recruited through local, compared to centralized or national, efforts [13]. Yet, it has been difficult to determine the most effective recruitment strategies and whether specific central efforts led to more successful local recruitment remains unclear [14], in part due to lack of prescreening data.

**Fig. 1:**
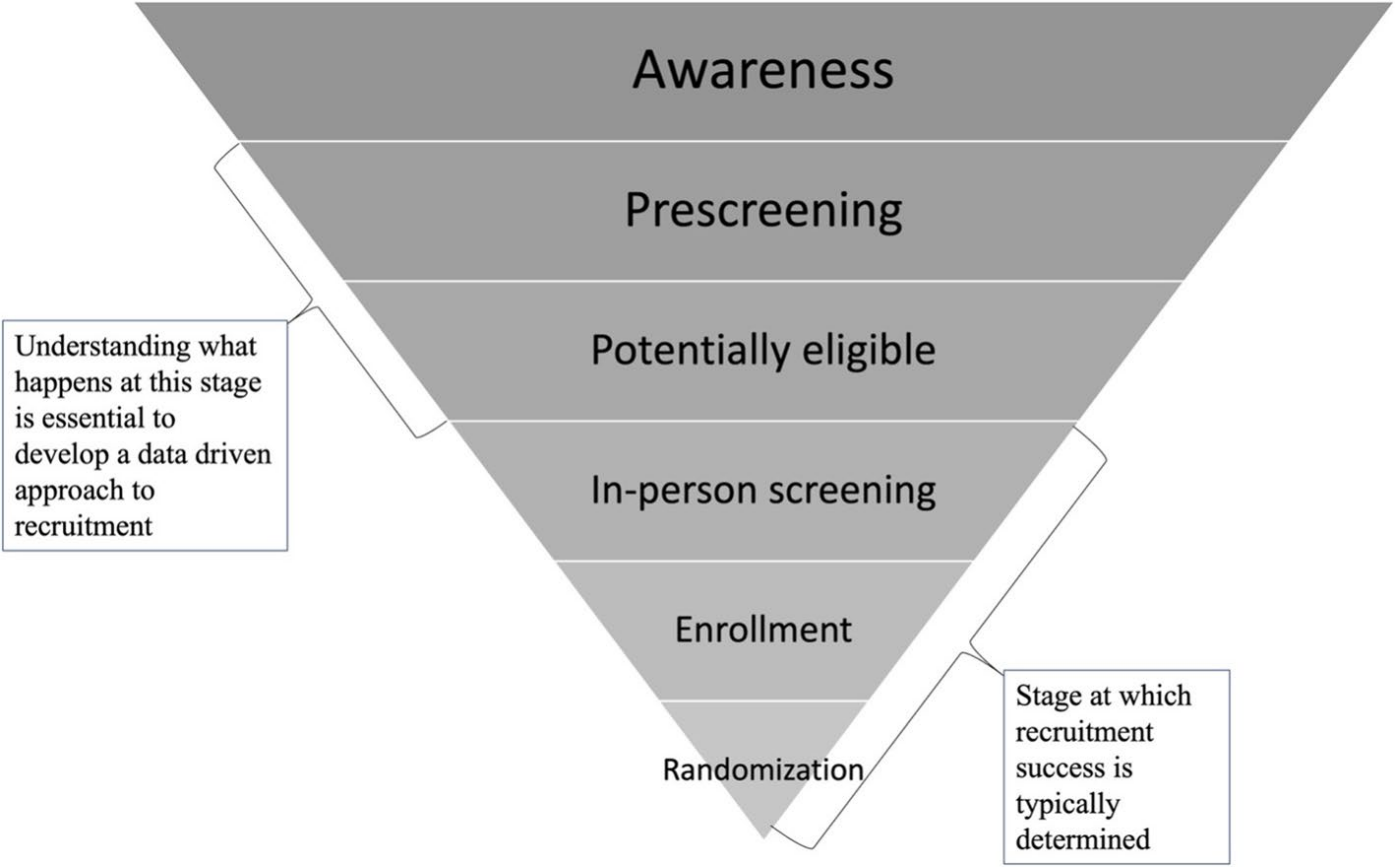
Clinical trial recruitment “funnel”

Systematizing and centralizing prescreening data capture is uncommon [15], in part due regulations that restrict formal data collection prior to consent and inclusion of prescreening data in parent trial databases as well as limited resources available to support this effort. Yet, to understand the effectiveness of recruitment strategies and potential sample bias, it is critical for trialists to assess the full recruitment process. This process begins with efforts to increase awareness and interest in trials and data are needed to examine this “top of the funnel” (Fig. 1). Limiting recruitment data to those collected after consent at in-person screening visits tells only part of the story.

To evaluate recruitment prior to enrollment, we designed and developed a centralized prescreening database, the data-driven approach to recruitment (DART), for an ongoing preclinical AD trial conducted by the NIA-funded Alzheimer’s Clinical Trials Consortium (ACTC). In this manuscript, we describe the design of the DART database and the share pilot data obtained from the initial vanguard phase used to assess the feasibility of centralizing prescreening data collection.

## Methods

The prescreening database was implemented under the AHEAD 3–45 study, a clinical trial evaluating the safety and efficacy of lecanemab (BAN2401, Eisai Inc.) in individuals who may be at increased biological risk for AD dementia [16, 17].

### Design

The DART database was collaboratively developed by a working group of ACTC coordinating center personnel and participating study sites. This working group met monthly from September 2020 to June 2021, first to design the data collection form and then to establish methods to minimize barriers and maximize the likelihood of adoption by many study sites. We viewed the inclusion of site personnel in this working group as essential to the success of this initiative.

### Variable selection

Variables selected for the DART database were aligned with the ACTC Minimal Data Set (MDS) recruitment and demographic variables (Table 1). To minimize site burden, we restricted the number of variables collected to eleven, including seven categorical and four free-text fields.

**Table 1:** Prescreening database variable description

**Variable**	**Age**	**Education**	**Race**	**Ethnicity**	**Occupation**	**Recruitment Source**		**Eligibility**
**Field Type**	Free text, integers only	Free text, integers only	Checkbox	Checkbox	Radio button	Checkbox		Yes/No, checkbox, free text
**Description**	User enters participant’s age	User enters participant’s years of education	• American Indian/Alaska Native • Asian ◦ Asian Indian ◦ Chinese ◦ Filipino ◦ Japanese ◦ Korean ◦ Vietnamese ◦ Other Asian ◦ Unknown or Not Reported • Black or African American • Hawaiian/other Pacific Islander • Caucasian or White • Other • Unknown/Not Reported	• Not Hispanic, Latino/a, or Spanish origin • Yes, Mexican, Mexican American, Chicano/a • Yes, Puerto Rican • Yes, Cuban • Yes, other Hispanic, Latino/a, or Spanish origin • Unknown/Not Reported	• Professional and Higher Executive Occupations, Chief Executives • Middle Professional Occupations/Small Business Owners • Managers • Support Personnel, Drafters, Technicians • Arts Design, Entertainment, Sports Occupations- Non-professional • Aids, Assistants, Clerks • Laborers • Other/Never Employed	• **National Campaign** ◦ Advertising ◦ Newspaper ◦ TV/Radio ◦ Other • **Social Media** ◦ Facebook ◦ Twitter ◦ Instagram ◦ YouTube ◦ Other • **Referral** ◦ Clinician at site ◦ Community clinician ◦ Community partner ◦ Participant ◦ NACC/other obs. ◦ Other	• **Registry** ◦ Local ◦ GeneMatch ◦ APT Webstudy ◦ Brain Health ◦ TrialMatch ◦ Other • **Local Campaign** ◦ Previous participant ◦ Local TV ◦ Local Radio ◦ Local theatre ◦ Outreach Event ◦ Other • **Website** ◦ Site website ◦ AHEAD website ◦ Clinicaltrials.gov ◦ Other ◦ TV/Radio • **Other**	• Yes ◦ Study ID# • No ◦ No longer interested ◦ No Study Partner ◦ MCI/AD Dx ◦ Medical exclusion (other than MCI/AD) ◦ No longer interested (concerns about investigational treatment ◦ No longer interested (concerns about radiation) ◦ Contraindications to MRI scanning • Other
**Variable**	**Sex**	**Zip Code**						
**Field Type**	Radio button	Free text, 5-digit integer						
**Description**	• Male • Female • Other	User enters participant’s 5-digit zip code						

### Site selection and responsibilities

In the DART vanguard phase, seven active AHEAD 3–45 sites collected prescreening data for approximately eight months. Vanguard sites were selected with attention to balance across site type and experience, including experience in similar trials, existing infrastructure to capture prescreening data, and existing prescreening databases. Vanguard sites were reimbursed for their participation.

A key component of the vanguard phase was a monthly meeting with representatives from each site to discuss implementation and to share and review metrics generated from prescreening data. The goal was to use preliminary site experiences to identify opportunities to improve database design, reduce site burden, facilitate timely data entry, and improve data integrity.

### Electronic data capture system (EDC)

We developed a separate EDC system specifically for this prescreening initiative, using the same framework as the AHEAD 3–45 study EDC [18]. Given that preexisting methods of capturing prescreen data varied widely across sites, we offered two options for sites to transmit prescreening data. For sites not capturing prescreening data electronically, data were entered directly into the EDC by site personnel. For sites with preexisting prescreening databases, batched upload was permitted, if it was performed at least every 2 weeks. A Data Transfer Agreement ensured the uploaded data were coded and formatted appropriately. Summary data reports were developed and distributed to sites and study leadership.

### Institutional review board approval and informed consent

The central IRB governing the AHEAD 3–45 study (Advarra, Columbia, MD) determined that the prescreening database was of minimal risk and did not require a formal informed consent process. Advarra granted the study a Waiver of Consent and Waiver of HIPAA after determining that the waiver satisfied the Common Rule and the criteria set forth in the HIPAA Privacy Rule at (45 CFR 164.512(i)(2)). Only deidentified information is collected in the central database.

### Statistical analysis

Time to contract execution and time to IRB approval for this initiative were defined as the time from when the site’s participation was confirmed to the time the contract was fully executed and central IRB approval was received by each site, respectively. Time to data entry was defined as the time the contract was fully executed to the time data entry/upload was initiated at the respective site.

Continuous variables were summarized by means and standard deviations while categorical variables were summarized using percentages. All statistical analyses were performed using R (Version 4.1.0).

## Results

Each of the seven vanguard sites was able to provide data in this initiative. Six of the sites opted to directly enter their data into the EDC, and one site utilized the batched upload functionality. Mean time to IRB approval after site selection was 124.1 days (range: 62–157 days), and mean time to contract execution was 213.1 days (range: 167–299 days). Mean time to data entry was 47.6 days (range: 2–128 days) across sites.

Sites reported some challenges while initiating this protocol at their sites. The most common barrier reported was the inability to identify staff at sites to complete the data entry, primarily affecting time to data entry. Other issues raised included the handling of incomplete records (i.e., participants decided not to proceed with screening before demographic information was collected), difficulty tracking prescreening status and updating records accordingly, and inconsistent entry of the PTID# in the prescreen EDC after eligible participants attended a study screening visit. For the batched upload site, minor formatting issues arose that required correction prior to incorporating the data into the database. These included using incorrect separators (“/” instead of “|”) and incorrect coding of gender. Once these barriers were addressed, subsequent issues entering the data were minimal.

Table 2 displays the demographic summaries and recruitment source of participants prescreened during the vanguard phase by site. Most prescreened participants in this vanguard phase self-reported as being female sex, White race, and non-Hispanic ethnicity. The total number of prescreened participants varied widely among the vanguard sites (range: 3–611 participants), as did the recruitment sources of the participants. Referrals through websites (including study website, site website, and ClinicalTrials.gov) consistently produced a meaningful proportion 55.8% (range: 36.0–75.0%) of prescreen activity.

**Table 2:** Demographics and recruitment source by site

	**Site 1** ***n* = 611**	**Site 2** ***n* = 17**	**Site 3** ***n* = 178**	**Site 4** ***n* = 118**	**Site 5** ***n* = 36**	**Site 6** ***n* = 66**	**Site 7** ***n* = 3**	**Total** ***n* = 1029**
**Race; *n* (%)**								
American Indian or Alaskan Native	3 (0.5)	0 (0.0)	1 (0.6)	3 (2.5)	1 (2.8)	0 (0.0)	1 (33.3)	9 (0.9)
Asian	4 (0.7)	1 (5.9)	14 (17.9)	0 (0.0)	2 (5.6)	1 (1.5)	0 (0.0)	22 (2.1)
Black or African American	36 (5.9)	0 (0.0)	4 (2.2)	2 (1.7)	1 (2.8)	1 (1.5)	0 (0.0)	44 (4.3)
Caucasian or White	465 (76.1)	16 (94.1)	155 (87.1)	106 (89.8)	30 (83.3)	49 (74.2)	2 (66.7)	823 (80.0)
Hawaiian or other Pacific Islander	1 (0.2)	0 (0.0)	1 (0.6)	0 (0.0)	1 (2.8)	0 (0.0)	0 (0.0)	3 (0.3)
Other	9 (1.5)	0 (0.0)	2 (1.1)	0 (0.0)	1 (2.8)	1 (1.5)	0 (0.0)	13 (1.3)
Unknown/not reported	99 (16.2)	0 (0.0)	1 (0.6)	8 (6.8)	0 (0.0)	14 (21.2)	0 (0.0)	122 (11.9)
**Ethnicity; *n* (%)**								
Not Hispanic/Latino/a/Spanish	461 (75.5)	17 (100.0)	156 (87.6)	109 (92.4)	34 (94.4)	49 (74.2)	3 (100.0)	829 (80.6)
Mex/Mex-American/Chicano/a	2 (0.3)	0 (0.0)	11 (6.2)	0 (0.0)	0 (0.0)	0 (0.0)	0 (0.0)	13 (1.3)
Puerto Rican	3 (0.5)	0 (0.0)	1 (0.6)	0 (0.0)	0 (0.0)	0 (0.0)	0 (0.0)	4 (0.4)
Cuban	1 (0.2)	0 (0.0)	1 (0.6)	0 (0.0)	0 (0.0)	0 (0.0)	0 (0.0)	2 (0.2)
Other Hispanic/Latino/a/Spanish	20 (3.3)	0 (0.0)	0 (0.0)	0 (0.0)	2 (5.6)	2 (3.0)	0 (0.0)	24 (2.3)
Unknown/not reported	124 (20.3)	0 (0.0)	9 (5.1)	9 (7.6)	0 (0.0)	15 (22.7)	0 (0.0)	157 (15.3)
**Gender; *n* (%)**								
Female	390 (63.8)	9 (52.9)	118 (66.3)	97 (82.2)	26 (72.2)	46 (69.7)	0 (0.0)	686 (66.7)
Male	184 (30.1)	8 (47.1)	60 (33.7)	21 (17.8)	10 (27.8)	20 (30.3)	2 (66.7)	305 (29.6)
Other	1 (0.2)	0 (0.0)	0 (0.0)	0 (0.0)	0 (0.0)	0 (0.0)	1 (33.3)	37 (3.6)
Unknown/prefer not to answer	36 (5.9)	0 (0.0)	0 (0.0)	0 (0.0)	0 (0.0)	0 (0.0)	0 (0.0)	1 (0.1)
**Age (years)**								
Mean (SD)	63.1 (7.2)	68.8 (7.2)	67.4 (8.1)	67.1 (6.7)	65.7 (6.9)	67.4 (6.8)	67.3 (8.3)	65.5 (7.6)
Missing (%)	354 (57.9)	0 (0.0)	0 (0.0)	22 (18.6)	0 (0.0)	22 (33.3)	0 (0.0)	398 (38.7)
**Hollingshead**								
Upper	62 (18.7)	1 (20.0)	32 (20.5)	14 (20.6)	10 (28.6)	8 (61.5)	2 (66.7)	129 (21.1)
Upper-middle	133 (40.1)	2 (40.0)	55 (35.3)	41 (60.3)	18 (51.4)	2 (15.4)	1 (33.3)	252 (41.2)
Middle	87 (26.2)	2 40.0)	37 (23.7)	8 (11.8)	5 (14.3)	2 (15.4)	0 (0.0)	141 (23.0)
Lower-middle	42 (12.7)	0 (0.0)	32 (20.5)	5 (7.4)	2 (5.7)	0 (0)	0 (0.0)	81 (13.2)
Lower	8 (2.4)	0 (0.0)	0 (0.0)	0 (0.0)	0 (0.0)	1 (7.7)	0 (0.0)	9 (1.5)
Missing	279	12	22	50	1	53	0	417
**Recruitment source; *n* (%)**^**a**^								
National campaign	5 (0.8)	2 (11.8)	8 (4.5)	3 (2.5)	0 (0.0)	0 (0.0)	0 (0.0)	18 (1.7)
Social media	2 (0.3)	1 (5.9)	20 (11.2)	2 (1.7)	1 (2.8)	4 (6.1)	0 (0.0)	30 (2.9)
Referral	27 (4.4)	3 (17.6)	9 (5.1)	27 (22.9)	8 (22.2)	12 (18.2)	2 (66.7)	88 (8.6)
Registry	15 (2.5)	1 (5.9)	42 (23.6)	3 (2.5)	1 (2.8)	15 (22.7)	0 (0.0)	77 (7.5)
Local campaign	252 (41.2)	0 (0.0)	33 (18.5)	8 (6.8)	0 (0.0)	4 (6.1)	0 (0.0)	297 (28.9)
Website	308 (50.4)	7 (41.2)	64 (36.0)	81 (68.6)	27 (75.0)	35 (53.0)	2 (66.7)	524 (50.9)
Missing	7 (1.2)	4 (23.5)	15 (8.4)	2 (1.7)	1 (2.8)	1 (1.52)	0 (0.0)	30 (2.9)

^a^Participants can provide more than one race and recruitment source, so total percentage may be > 100%

Table 3 summarizes recruitment source data by race and ethnicity, respectively. Websites were the most common recruitment source across racial groups. Hispanic participants appeared to have more often been recruited from registries and local recruitment efforts than to non-Hispanics participants.

**Table 3:** Recruitment source by race and ethnicity

	**National campaign**	**Social media**	**Referral**	**Registry**	**Local campaign**	**Website**	**No referral source/missing**
**Race; *n* (%)**							
American Indian or Alaskan Native	0 (0)	0 (0.0)	2 (22.2)	0 (0.0)	1 (11.1)	8 (66.7)	0 (0.0)
Asian	1 (4.5)	2 (9.1)	2 (9.1)	3 (13.6)	7 (31.8)	10 (45.5)	1 (4.5)
Black or African American	1 (2.3)	0 (0.0)	5 (11.4)	1 (2.2)	13 (29.5)	23 (52.3)	1 (2.3)
Caucasian or White	14 (1.7)	27 (3.3)	73 (8.9)	54 (6.6)	241 (29.3)	420 (51.0)	24 (2.9)
Hawaiian or other Pacific Islander	0 (0.0)	1 (3.3)	0 (0.0	0 (0.0)	1 (33.3)	1 (33.3)	0 (0.0)
Other	0 (0.0)	0 (0.0)	1 (7.7)	2 (15.4)	8 (61.5)	2 (14.5)	0 (0.0)
Unknown/not reported	2 (1.6)	0 (0.0)	6 (4.9)	17 (13.9)	28 (23.0)	66 (54.1)	4 (3.3)
**Total**	18 (1.7)	30 (2.9)	88 (8.6)	77 (7.5)	298 (28.9)	524 (50.9)	30 (2.9)
**Ethnicity; *n* (%)**							
Not Hispanic/Latino/a/Spanish	14 (1.7)	26 (3.1)	77 (9.3)	50 (6.0)	250 (30.2)	419 (50.5)	26 (3.1)
Any/All Hispanic/Latino/a/Spanish	2 (4.7)	2 (4.7)	3 (7.0)	6 (14.0)	16 (37.2)	15 (34.9)	0 (0.0)
Unknown/not reported	2 (1.3)	2 (1.3)	8 (5.1)	21 (13.4)	31 (19.8)	90 (57.3)	4 (2.6)
**Total**	18 (1.8)	30 (2.9)	88 (8.6)	77 (7.5)	297 (28.9)	524 (50.9)	30 (2.9)

Participants can provide more than one recruitment source, so total percentage may be > 100%

Table 4 presents eligibility results, reported by race and ethnicity. During the vanguard phase, 19% of prescreened participants had been deemed eligible for in-person screening, though many prescreened participants remained in the prescreening process at the time of data freeze.

**Table 4:** Eligibility by race and ethnicity

	**Eligible**	**Ineligible**	**Not yet entered**	**Total**
**Race; *n* (% of total)**				
American Indian or Alaskan Native	5 (55.6)	1 (11.1)	3 (33.3.4)	9
Asian	7 (31.8)	10 (45.5)	5 (22.7)	22
Black or African American	4 (9.1)	17 (38.6)	23 (52.3)	44
Caucasian or White	181 (22.0)	256 (31.1)	386 (46.9)	823
Hawaiian or other Pacific Islander	0 (0.0)	1 (33.3)	2 (66.7)	3
Other	2 (15.4)	5 (38.4)	6 (46.2)	13
Unknown/not reported	3 (2.5)	39 (32.0)	80 (65.6)	122
**Total**	200 (19.4)	327 (31.8)	502 (48.8)	1029
**Ethnicity; *n* (% of total)**				
Not Hispanic/Latino/a/Spanish	191 (23.0)	264 (31.8)	374 (45.2)	830
Mex/Mex-American/Chicano/a	2 (15.4)	3 (23.1)	8 (61.5)	13
Puerto Rican	1 (25.0)	0 (0.0)	3 (75.0)	4
Cuban	0 (0.0)	0 (0.0)	2 (100.0)	2
Other Hispanic/Latino/a/Spanish	1 (4.2)	7 (29.2)	16 (66.7)	24
Unknown/not reported	5 (3.2)	53 (33.8)	99 (63.1)	157
**Total**	200 (19.4)	327 (31.8)	502 (48.8)	1029

Participants can have more than one race; the sum (%) may exceed 100% or total number of participants, respectively

Table 5 shows the distribution of reasons why participants did not continue to in-person screening by race. The most frequent reason was a loss of interest or concern about study burden, though many participants (50%) who did not proceed to in person screening had reason entered as “other.”

**Table 5:** Reason for prescreen fail by race

	**American Indian or Alaskan Native**	**Asian**	**Black or African American**	**Caucasian or White**	**Other**	**Unknown or not reported**	**Hawaiian or other Pacific Islander**	**Total**
No longer interested—concerns about burden/duration	1 (100%)	1 (10.0%)	3 (17.6%)	65 (25.4%)	1 (20.0%)	7 (18.0%)	0 (0.0%)	78 (23.9%)
No study partner	0 (0.0%)	1 (10.0%)	1 (5.9%)	7 (2.7%)	1 (20.0%)	0 (0%)	0 (0.0%)	9 (2.8%)
MCI/AD diagnosis	0 (0.0%)	0 (0.0%)	0 (0%)	8 (3.1%)	0 (0.0%)	0 (0%)	0 (0.0%)	8 (2.5%)
Medical exclusion (other than MCI/AD diagnosis)	0 (0.0%)	1 (10.0%)	3 (17.7%)	44 (17.2%)	0 (0.0%)	2 (5.1%)	1 (100.0%)	51 (15.6%)
No longer interested—concerns regarding investigational treatment	0 (0.0%)	0 (0.0%)	0 (0.0%)	10 (3.9%)	0 (0.0%)	2 (5.1%)	0 (0.0%)	12 (3.7%)
No longer interested—concerns regarding radiation	0 (0.0%)	0 (0.0%)	0 (0.0%)	3 (1.2%)	0 (0.0%)	0 (0%)	0 (0.0%)	3 (0.9%)
Contraindications to MRI scanning	0 (0.0%)	0 (0.0%)	2 (11.8%)	8 (3.1%)	0 (0.0%)	1 (2.6%)	0 (0.0%)	11 (3.4%)
Other	0 (0.0%)	7 (70.0%)	8 (47.1%)	121 (47.3%)	4 (80.0%)	28 (71.8%)	0 (00%)	167 (51.1%)

Participants can have more than one reason for prescreen fail and race; the sum (%) may exceed 100% or total number of participants, respectively

Qualitative feedback collected from the vanguard sites through the monthly meetings suggested that the metrics routinely shared were helpful in guiding local recruitment efforts. Therefore, site-specific reports will be generated for all participating sites, and study-level web reports will be made available in real-time to study leadership for the study-wide implementation phase of this initiative.

## Discussion

Through the DART initiative, we demonstrated that the collection of prescreening data in a multi-site clinical trial is feasible. Seven vanguard sites were able to enter or upload prescreening data into an EDC developed specifically for this purpose. We also demonstrated that meaningful questions can be answered by capturing key variables from the prescreening phase.

From these preliminary data, we identified recruitment strategies that more often yielded prescreened participants than others. We also observed early trends that the effectiveness of recruitment sources may differ among racial and ethnic groups. For example, multiple sources of outreach including local campaigns, such as local television or radio interviews, account for a slightly higher percentage of Hispanic prescreens, compared to non-Hispanic, though the sample size remains low. Moreover, the AHEAD 3–45 study website accounted for a high percentage of prescreened participants across several racial and ethnic groups. Given that most of the central recruitment strategies implemented for the study promoted the study website, this suggests that these efforts may be a critical element toward improving demographic representation in this study.

Measuring prescreen failure rates may offer important guidance to trial leadership. From these vanguard sites, we found that losing interest and the unwillingness to endure trial burden were more frequent reasons for people to not proceed to in-person screening than was ineligibility based on trial enrollment criteria. This finding could help inform changes in recruitment materials or site practices to explore means to reduce burden or to make research participation more appealing to potential participants. Notably, had trial enrollment criteria been a primary reason for failure to advance to in-person screening, such data would provide the study team the opportunity to review and potentially revise the trial inclusion/exclusion criteria.

### Next steps

As we move towards study-wide implementation phase of DART, some changes have been made to the data collection form. As noted above, a high percentage of reasons participants prescreen fail were entered as “other,” followed by a free-text description. In response to this, we expanded the options that sites can select for “Reason for Prescreen Fail.” We used the reasons written in the free-text field to expand the categories to match the exact inclusion/exclusion criteria from the trial, including “age,” “does not have additional risk factor (< 65 years old only),” “already enrolled in another clinical trial,” “no longer interested—lives too far from study site,” and “lost to follow-up/unable to contact.” The addition of these options will help limit the number of “other” reasons in the expanded initiative and permit more useful and analyzable data. We also decided to eliminate the collection of occupation and education as these variables were infrequently collected in the initial stages of the prescreening process and hence resulted in substantial missing data.

Using these methods to centralize prescreening data collection requires effort from site personnel, project management, data management, and biostatistics, making funding an essential component for success. Ideally, resources to collect prescreening data would be included in the original study budget. Some but not all trials offer start-up funds for the effort of securing IRB approval and other preparatory needs, as well as recruiting participants for initial screens. Inclusion of the site effort to put a prescreening database and infrastructure in place might ideally be included as a line-item in start-up budgets. Alternatively, the resources for maintaining prescreening databases might be provided as part of infrastructure resources for new or established trial site consortia.

### Limitations

We acknowledge some important limitations. The DART initiative vanguard phase utilized only 7 sites from the AHEAD 3–45 study with a small number of data variables. This was done to limit site, participant, and coordinating burden and to enable collection of preliminary experiences with the initiative. The prescreening initiative may result in duplication of effort for at least some sites that already capture prescreening data electronically, though we offered the batched upload option to minimize burden for those sites. The initiative has costs, which may limit the ability for small and/or underfunded trials and trial networks to create this infrastructure, potentially limiting the generalizability of this effort. Alternatively, collecting these data may enable efficient use of recruitment resources, potentially reducing overall trial costs. The initiative started early in the recruitment phase of the AHEAD 3–45 study, which may have had an impact on the number of prescreens sites entered. This may accurately reflect start-up in future trials, but we have limited information related to the main stages of study accrual. The COVID-19 pandemic may also have influenced these results, given the impact on site staffing during the vanguard phase and possible effects on willingness to participate in the AHEAD 3–45 study. Finally, though the study website yielded the most prescreens, it is unclear how participants found the study website as other advertisements may have directed them towards the website. Though this is a limitation, it does support the utility of a study website as a mechanism for potential participants to connect with sites.

## Conclusions

Recruitment for clinical trials is challenging and time consuming. Relying on post-consent screening data is insufficient to fully capture the effectiveness of centralized and local efforts to accrue a full sample and identify sources of selection bias. The centralized collection of prescreening data may increase the efficiency, speed, and effectiveness of study recruitment, including enrolling a cohort more representative of the population at large. The vanguard phase of this innovative prescreening database initiative demonstrated the feasibility of establishing such a database and allowed the project team to learn important lessons to increase the likelihood of a successful study-wide implementation.

## Data Availability

Not applicable.

## Abbreviations

NIA: National Institute on Aging
ACTC: Alzheimer’s Clinical Trials Consortium
AD: Alzheimer’s disease
A4 Study: Anti-Amyloid Treatment in Asymptomatic AD study
DART: Data-driven approach to recruitment
EDC: Electronic data capture system
PTID: Participant ID

## References

[1] Schneider LS (2012). Recruitment methods for United States Alzheimer disease prevention trials. J Nutr Health Aging.

[2] Vellas B, Hampel H, Rouge-Bugat ME, Grundman M, Andrieu S, Abu-Shakra S (2012). Alzheimer’s disease therapeutic trials: EU/US Task Force report on recruitment, retention, and methodology. J Nutr Health Aging.

[3] Kasenda B, von Elm E, You J, Blumle A, Tomonaga Y, Saccilotto R (2014). Prevalence, characteristics, and publication of discontinued randomized trials. JAMA.

[4] Gilmore-Bykovskyi AL, Jin Y, Gleason C, Flowers-Benton S, Block LM, Dilworth-Anderson P (2019). Recruitment and retention of underrepresented populations in Alzheimer’s disease research: a systematic review. Alzheimers Dement (N Y).

[5] Nuno MM, Gillen DL, Dosanjh KK, Brook J, Elashoff D, Ringman JM (2017). Attitudes toward clinical trials across the Alzheimer’s disease spectrum. Alzheimers Res Ther.

[6] FDA (2020). Enhancing the diversity of clinical trial populations - eligibility criteria, enrollment practices, and trial designs guidance for industry - guidance document.

[7] Grill JD, Sperling RA, Raman R (2022). What should the goals be for diverse recruitment in Alzheimer clinical trials?. JAMA Neurol.

[8] Oh SS, Galanter J, Thakur N, Pino-Yanes M, Barcelo NE, White MJ (2015). Diversity in clinical and biomedical research: a promise yet to be fulfilled. PLoS Med.

[9] Wendler D, Kington R, Madans J, Van Wye G, Christ-Schmidt H, Pratt LA (2006). Are racial and ethnic minorities less willing to participate in health research?. PLoS Med.

[10] Grill JD, Galvin JE (2014). Facilitating Alzheimer disease research recruitment. Alzheimer Dis Assoc Disord.

[11] Sperling RA, Karlawish J, Johnson KA (2013). Preclinical Alzheimer disease-the challenges ahead. Nat Rev Neurol.

[12] Sperling RA, Rentz DM, Johnson KA, Karlawish J, Donohue M, Salmon DP (2014). The A4 study: stopping AD before symptoms begin?. Sci Transl Med..

[13] Raman R, Quiroz YT, Langford O, Choi J, Ritchie M, Baumgartner M (2021). Disparities by race and ethnicity among adults recruited for a preclinical Alzheimer disease trial. JAMA Netw Open.

[14] Tarrant SD, Bardach SH, Bates K, Nichols H, Towner J, Tamatha C (2017). The Effectiveness of small-group community-based information sessions on clinical trial recruitment for secondary prevention of Alzheimer’s disease. Alzheimer Dis Assoc Disord.

[15] NIA/NIH. Alzheimer’s Disease and Related Dementias Clinical Studies Recruitment Planning Guide 2019 [Available from: https://www.nia.nih.gov/sites/default/files/2019-05/ADEAR-recruitment-guide-508.pdf.

[16] [Available from: https://clinicaltrials.gov/ct2/show/NCT04468659.

[17] Rafii MS, Sperling RA, Donohue MC, Zhou J, Roberts C, Irizarry MC (2022). The AHEAD 3–45 Study: design of a prevention trial for Alzheimer’s disease. Alzheimers Dement.

[18] Jimenez-Maggiora GA, Bruschi S, Qiu H, So JS, Aisen PS (2022). Corrigendum to: ATRI EDC: a novel cloud-native remote data capture system for large multicenter Alzheimer’s disease and Alzheimer’s disease-related dementias clinical trials. JAMIA Open..

